# Exercise: A Protective Measure or an “Open Window” for COVID-19? A Mini Review

**DOI:** 10.3389/fspor.2020.00061

**Published:** 2020-05-15

**Authors:** João B. Ferreira-Júnior, Eduardo D. S. Freitas, Suene F. N. Chaves

**Affiliations:** ^1^Federal Institute of Sudeste of Minas Gerais, Campus Rio Pomba, Rio Pomba, Brazil; ^2^Neuromuscular Laboratory, Department of Health and Exercise Science, University of Oklahoma, Norman, OK, United States; ^3^Department of Sports, Federal University of Minas Gerais, Belo Horizonte, Brazil

**Keywords:** coronavirus, immune system, endurance training, resistance training, blood flow restriction, no load resistance training

## Abstract

The coronavirus disease 2019 (COVID-19) has spread to at least 115 countries and caused an alarming number of deaths. The current outbreak has lead authorities from many countries to adopt several protective measures, including lockdown and social distancing. Although being a reasonable measure to counteract the COVID-19 contamination, the restrictive measures have limited individual's ability to perform exercise outdoors or in gyms and similar facilities, thus raising the risks for chronic health conditions related to a sedentary lifestyle. The recent exercise recommendations to counteract the potential deleterious effects of COVID-19-related lockdown have not fully addressed resistance exercise interventions as potential home-based exercise strategies. Additionally, the following questions have been constantly raised: (1) Is training status capable of protecting an individual from COVID-19 infection?; and (2) Can a single endurance or resistance exercise session acutely increase the risks for COVID-19 infection? Therefore, the current mini review aimed to focus on these two concerns, as well as to discuss the potential use of practical blood flow restriction and no load resistance training as possible resistance exercise strategies that could be performed during the current COVID-19 pandemic.

## Introduction

The coronavirus disease 2019 (COVID-19) outbreak was initially restricted to the continental China area, but it quickly gained international proportions spreading to at least 114 additional countries (Holshue et al., 2020). This caused the World Health Organization to declare it a pandemic on March 11th, 2020, the first since the H1N1 2009 pandemic.

As of May 3rd, 2020, the World Health Organization had reported 3,349,786 confirmed cases of the disease worldwide, with a total of 193,710 deaths (World Health Organization, 2020). The current outbreak has lead authorities from many countries to adopt several protective measures, including lockdown and social distancing (Wang et al., 2020). Despite being a reasonable measure in the face of the potential consequences of COVID-19 contamination, social lockdown may negatively impact physical and mental health (Chen et al., 2020; Zhu, 2020). It may reduce daily activity levels and increase sedentary behaviors, thus raising the risks for chronic health conditions related to a sedentary lifestyle. In this sense, recent recommendations have highlighted the importance of maintaining exercise routines during this pandemic period (Chen et al., 2020; Zhu, 2020), with simple and feasible home-based exercise modalities being considered as potential training strategies to be performed to counteract the deleterious effects of lockdown and social distancing (Chen et al., 2020).

COVID-19 is caused by a coronavirus, a large group of single-stranded RNA viruses that infects both humans and animals. In humans, COVID-19 seems to target primarily the respiratory system and can be manifested through a variety of symptoms or may be asymptomatic in some cases. The most common symptoms of the disease include rhinorrhea, sore throat, sneezing, cough, fever, muscle soreness, and fatigue (Rothan and Byrareddy, 2020; Wu et al., 2020). In more severe cases, the symptoms may evolve to RNAemia, acute respiratory distress syndrome, acute cardiac injury, and incidence of ground-glass opacities, eventually leading to death (Rothan and Byrareddy, 2020). Patients infected with COVID-19 have also presented immune system alterations commonly observed in upper respiratory tract infections (URTI), such as higher leukocyte numbers and high blood levels of cytokines and chemokines, interleukins, monocyte chemoattractant proteins, and macrophage inflammatory proteins (Huang et al., 2020; Rothan and Byrareddy, 2020). It has been demonstrated that the rate of recovery from COVID-19 seems to be affected by age and status of the patient's immune system (Rothan and Byrareddy, 2020). Additionally, several studies have suggested that exercise training may impact the immune system by increasing leukocytes, and reducing inflammatory cytokines and chemokines (Nieman et al., 1989, 1993; Matthews et al., 2002; Chaouachi et al., 2008). On the other hand, endurance or resistance exercise sessions may acutely cause a condition of immunosuppression by reducing both lymphocytes and natural killer cells (Nieman et al., 1995; Pedersen and Bruunsgaard, 1995; Kakanis et al., 2010).

In this context, endurance and resistance exercise interventions have not been fully addressed as potential home-based exercise strategies to counteract the potential deleterious effects of lockdown and social distancing. In order to be performed at home, exercise modalities should require little, to no equipment, do not require a spotter, and be easy to perform. Therefore, resistance exercise modalities such as practical blood flow restriction (BFR) and no load resistance training (NLRT) meet these requirements and may serve as potential exercise strategies to be performed at home.

Thus, in the face of the current situation, the following two questions have been constantly asked: (1) Is training status capable of protecting from COVID-19 infection?; and (2) Can a single endurance or resistance exercise session acutely increase the risks for COVID-19 infection? Although relevant, both questions have not been adequately addressed in any of the recently released COVID-19 exercise recommendations (Chen et al., 2020; Zhu, 2020). Therefore, the current mini review aimed to address these concerns, as well as to discuss the potential use of practical BFR resistance training and NLRT as possible resistance exercise interventions that could be performed during the current COVID-19 pandemic.

## Can Training Status Protect Against COVID-19 Infection?

The relationship between training status and protective responses against upper respiratory tract infections (URTI) has been an active area of research over the past decades. It has been argued that regular exercise decreases blood circulation of inflammatory cytokines, decreases oxidative stress, and improves function of various immune cells in the resting state, which would potentially reduce the risks for URTI cases (Peters, 1997; Abd El-Kader and Al-Shreef, 2018; Aoi and Naito, 2019). From a clinical perspective, one of the most useful parameters of immune function is the incidence of URTI (Aoi and Naito, 2019), thus, we decided to focus on this parameter. Heath et al. (1991) reported an URTI incidence of 1.2 per runner per year, however, higher URTI incidences have been reported in previous studies in untrained subjects; 2.3–4.1 URTI per person per year (Gwaltney et al., 1966; Fox et al., 1972). Additionally, there is a number of studies suggesting that training status may protect against URTI due to a possible improvement in immune function parameters, such as increased lymphocytes, leucocytes, and killer cell counts, and others (Nieman et al., 1989, 1993; Matthews et al., 2002; Chaouachi et al., 2008).

For instance, Nieman et al. (1989) evaluated 273 runners over the course of 2 months of training prior to a 5 km, a 10 km, and a half-marathon competition. The runners preparing for a half-marathon presented lower URTI cases (6.8%) than runners who prepared for the 5 and 10 km races (17.9%). Moderate levels of physical activity have also been associated with a reduced risk of URTI cases in comparison to sedentary individuals (Matthews et al., 2002). A previous study examined URTI incidences in elderly women who completed 12 weeks of walking (*n* = 14) or calisthenic (*n* = 16) training programs, and elderly women endurance runners (*n* = 12), recruited for a cross-sectional comparison (Nieman et al., 1993). The highest URTI incidence was observed in the calisthenic group (50%), followed by the walker group (21.4%), while the highly conditioned endurance running group displayed the lowest rate (8.3%). These incidence rates seemed to be considerably higher than expected, and a further detailed examination of the study design revealed that it was conducted during the fall season. As mentioned by the authors, a symptomatology analysis of each subject enrolled in the study revealed that only one subject developed URTI due to flu, whereas the remaining 11 URTI cases were reported as common colds (Nieman et al., 1993). Additionally, it is surprising that the highly conditioned group presented a body mass index similar to that of young females, and displayed natural killer cell activity levels greater than the walking and calisthenic groups, and even superior to young adult women. Additionally, the authors hypothesized that elderly women not engaged in cardiorespiratory exercise programs would be more susceptible to URTI, which is supported by a recent study showing that endurance training elicits improvements in both immune parameters and in inflammatory markers when compared to resistance exercise in older individuals (Abd El-Kader and Al-Shreef, 2018).

On the other hand, other studies have shown no protective effects of exercise training programs on URTI incidences (Nieman et al., 1990b; Kostka and Prączko, 2007; Kostka et al., 2008; Walsh et al., 2011). To illustrate, a study with obese sedentary women who completed 15 weeks of endurance training (walking at 60% heart rate reserve), five times a week, reported no differences in the number of URTI in comparison to the untrained control subjects (Nieman et al., 1990b). However, the number of days with URTI symptoms was lower in the exercise group in comparison to the control group, which was confirmed by additional studies (Kostka and Prączko, 2007; Kostka et al., 2008; Walsh et al., 2011). An additional study did not find any relationship between perceived physical fitness levels and URTI incidences (Kostka et al., 2008).

Conversely, an increased rate of infections among athletes in comparison to recreational runners was observed after the 2000 Stockholm Marathon, which was attributed to the higher training volume performed by the athletes (Ekblom et al., 2006). Additional recent studies demonstrated an increase in URTI symptoms during the competitive season or during periods of excessive stress from training (Ferrari et al., 2013; Brunelli et al., 2014). It is important to highlight that an imbalance between training stimulus and recovery state may contribute to overreaching, overtraining syndrome and illnesses, including infectious diseases (Kellmann et al., 2018), which would contribute to the increase in URTI incidences among athletes. Moreover, there appears to be a difference in infection rates among athletes of different categories (i.e., recreational vs. national vs. international athletes; Walsh and Oliver, 2016). The greater training volume performed by athletes can up-regulate the T-helper cell profile, leading to cell-mediated immunity, consequently predisposing athletes to allergic reactions, and greater frequency of reported URTI such as allergic rhinitis (Smith, 2003; Robson-Ansley et al., 2012). Lastly, athletes may also be more likely to experience URTI related to allergies rather than infectious processes (Robson-Ansley et al., 2012).

The contradictions displayed across the studies discussed above may be related to differences in the immune response to training status (more trained vs. less trained individuals). Some studies have reported reductions in the lymphocyte proliferative response (Papa et al., 1989) and suppressed neutrophil function (Lewicki et al., 1988; Baj et al., 1994), whereas other studies have shown no alteration in either lymphocyte or neutrophil status after a period of exercise (Tvede et al., 1991; Flynn et al., 1999; Ferrari et al., 2013), or even increases (Brunelli et al., 2014) when comparing different training statuses. These conflicting results may be due to differences in research design across studies, such as: (1) cross-sectional versus longitudinal studies; (2) exercise intensity and exercise volume; (3) training status (sedentary individuals; recreational-, national- or international-level athletes); (4) the immune system parameters assessed; and (5) genetic influences on URTI (Pedersen and Hoffman-Goetz, 2000; Walsh and Oliver, 2016; Peake et al., 2017). Additionally, most of the available studies are based on self-reported URTI symptoms (Pedersen and Hoffman-Goetz, 2000), and should be considered with some caution (Spence et al., 2007; Walsh and Oliver, 2016). There are also a limited number of studies that have measured immune function parameters and URTI incidences simultaneously across distinct exercise training programs (Ferrari et al., 2013; Brunelli et al., 2014); and as such, a causal relationship between exercise training immunity and infection have been only speculated (Cavaglieri et al., 2011; Walsh and Oliver, 2016).

Therefore, considering the available scientific evidences, it remains unclear if training status affects URTI incidence, and it cannot be concluded that trained individuals are more protected against COVID-19 infection when compared to untrained individuals. Additionally, the COVID-19 pandemic has provided an opportunity to evaluate the exercise training history of contaminated patients, in order to provide data for further investigations on the protective effects of training status against URTI.

## Can an Acute Exercise Session Increase the Risks for COVID-19 Infection?

The paradigm that a single exercise session may acutely increase the risks of viral or bacterial infections has commonly been referred to as the “open window” theory and represents a condition of immunosuppression (i.e., lymphocytes reduction and suppressed function of natural killer cells) occurring after prolonged high-intensity exercise (Pedersen and Bruunsgaard, 1995; Kakanis et al., 2010). Additionally, exercise-induced muscle damage may activate immune cells because of the unaccustomed or high-intensity exercise sessions needed to promote skeletal muscle regeneration (Clarkson and Dedrick, 1988; Paulsen et al., 2012; Ferreira-Junior et al., 2014; Peake et al., 2017). Leukocytes seem to be mobilized by sarcomere disruption following several muscle contractions accompanied by the production of inflammatory cytokines and reactive oxygen species (Clarkson and Dedrick, 1988; Paulsen et al., 2012; Ferreira-Junior et al., 2014; Peake et al., 2017). This process is followed by a short-term reduction in muscular strength and increased localized muscle swelling, delayed-onset muscle soreness, and impaired motor skill learning (Ferreira-Junior et al., 2015; Leite et al., 2019).

Several studies have investigated the effects of a single exercise session on immune function by measuring acute changes in various parameters (Flynn et al., 1999; Fahlman et al., 2000; Steensberg et al., 2001; Kakanis et al., 2010). However, few studies have utilized resistance exercise protocols (Nieman et al., 1995; Flynn et al., 1999) or home-based exercises, those likely to be performed during the current pandemic which most likely consist of adapted forms of resistance exercise. Thus, it is important to explore the implications of acute sessions of resistance exercise on the immune function and whether it may potentially increase the risks for COVID-19 infection by causing immunosuppression.

Nieman et al. (1995) had participants perform multiple sets of squat exercise to failure at 65% of one-maximum repetition (1-RM) and observed conflicting results with leukocytes counts increasing immediately post-exercise and remaining elevated up to 2 h after, whereas lymphocytes number increased post-exercise but decreased below baseline levels 2 h post-exercise. Nonetheless, it is important to note that Nieman et al. (1995) utilized a sample of 10 resistance trained individuals with a mean age of ~25 years, however, COVID-19 is particularly dangerous to older individuals, which represents most of the COVID-19-related deaths reported to date (Verity et al., 2020). Therefore, it is important to consider the potential immunosuppressive effects of resistance exercise for elderly participants. In this regard, (Flynn et al., 1999) investigated the acute effects of a resistance exercise session performed at 80% 1-RM on several immune function parameters in women aged 67 and 84 years and reported no suppression of immune function during the recovery period from the exercise bout. Finally, it is important to highlight that URTI incidences were not assessed in any of these studies.

Regarding endurance exercise, there were no increase in infection rates following 5 km, 10 Km, or half-marathon races when compared to the week prior to the race (Nieman et al., 1989). Similar results were observed in 1,694 runners who finished the 2000 Stockholm Marathon (Ekblom et al., 2006). On the other hand, Nieman et al. (1990a) observed that 12.9% of 1,828 participants of the 1,987 Los Angeles Marathon reported infection episodes during the week following the marathon in comparison to only 2.2% of the 134 similarly trained runners who did not participate in the event.

The different results across these studies may be due to the variability in the immune system responses to an acute exercise session. Although a number of studies have observed an immunosuppressive response after prolonged and intense exercise bouts (Steensberg et al., 2001; Kakanis et al., 2010), no changes in immune function have also been observed (Flynn et al., 1999). Another study did not report alterations in T helper-2 lymphocytes after 2.5 h of endurance exercise performed at 75% of maximal oxygen consumption in trained runners (Steensberg et al., 2001), suggesting that there was no immunosuppression post-exercise (Smith, 2003). The acute immune response to exercise also appears to depend on exercise volume and intensity. Moderate endurance exercise (<2 h, at lactate steady state near 2 mmol.l^−1^, or <30 min at a lactate steady state of 4 mmol.l^−1^) may yield smaller changes in immune function than strenuous exercise (~100% anaerobic threshold or above, or >2 h of exhaustive endurance exercise; Gabriel and Kindermann, 1997). Additionally, whenever an acute endurance exercise section caused an immunosuppressive response, it was considered relatively small (Gabriel and Kindermann, 1997).

A recent study evaluated 117 runners who complete the 2010 London Marathon, it was reported that 19% of the recreational runners reported URTI, while 45% of the athletes displayed URTI at some point in the 15 days following the marathon (Robson-Ansley et al., 2012). It was also reported that 58% of the runners who presented URTI were allergic rather than infectious in nature. Interestingly, the marathon took place during the pollen season, which exposes athletes to allergens (Robson-Ansley et al., 2012). Hence, this study indicates that further rigorously controlled scientific researches on this topic is required in order to minimize possible sources of bias (e.g., exercise intensity and volume, training status, measurement of immunological and pathogen parameters, etc.).

Therefore, considering the current scientific evidence, the “open window” theory and the hypothesis that an exercise session would acutely increase the risk of COVID-19 infection remains speculative. However, as mentioned earlier, a strenuous exercise session (intensity higher than 100% of anaerobic threshold, or 2–3 h of exhaustive endurance exercise) might evoke greater immunodepression (Gabriel and Kindermann, 1997). Thus, individuals are recommended to perform short exercise sessions (≤1.5 h) at moderate or low intensity. Physicians are also recommended to include in the patient record if any exercise session was performed prior to the COVID-19 infection in order to provide further data to examine the “open window” theory.

## NLRT and BFR Resistance Training as Home-Based Exercise Interventions

NLRT is a relatively novel resistance training strategy that consists of repeatedly contracting a muscle or muscle group as hard as possible through a full range of motion without the use of an external load (Counts et al., 2016; Gentil et al., 2017). It can be easily performed by any individual, including hospitalized patients (Barbalho et al., 2017). A typical NLRT session consists of 4 sets of 20 repetitions with 30 s of rest between sets (Counts et al., 2016) for several exercises (e.g., squats, and arm press or push exercises). A recent study reported that this training strategy resulted in an increase in muscle size, measured by ultrasound, similar to traditional high load resistance training (Counts et al., 2016). Strength gains were also reported, although at lower levels when compared to traditional high load resistance training.

Another training modality that may be utilized by those who desire to remain physically active is low-load resistance exercise combined with BFR. This training method has been considered a relatively safe and effective training strategy across different populations to increase muscle size and strength (Kubo et al., 2006; Patterson et al., 2019). BFR resistance exercise is performed with the aid of pneumatic cuffs placed at the most proximal portion of the arms or legs, inflated to a target pressure, and deflated at the end of the exercise bout. Although the efficacy of this most traditional form of BFR resistance exercise has been extensively reported in the literature, it still requires expensive equipment, which makes it difficult for it to be performed at home. Therefore, a more feasible alternative has been developed, which has been termed practical BFR resistance exercise. Practical BFR consists of utilizing elastic wraps to reduce blood flow, rather than the traditional restrictive cuffs, which makes practical BFR resistance exercise much more accessible to the general population and easy to perform (Wilson et al., 2013; Lowery et al., 2014).

The general recommendation is to perform a first set of 30 repetitions followed by three additional sets of 15 repetitions using low-loads (20–50% of 1-RM), and 30 to 60 s of rest interval between sets (Patterson et al., 2019). This protocol can be performed utilizing squats, arm or leg curls, or any other exercise that utilizes the arms or legs. The restrictive pressure applied using the elastic wraps should be enough to allow the recommend number of sets and repetitions to be completed. Full details regarding practical BFR training are described elsewhere (Wilson et al., 2013; Lowery et al., 2014). Practical BFR resistance exercise may cause local discomfort but should not elicit strong pain. If strong pain is perceived, the elastic wraps should be loosened to a more comfortable level. This issue may be avoided by tightening the wrap to a perceived tightness of 7 out of 10 in the perceived pressure scale (Wilson et al., 2013) to achieve the desired physiological stress. Recent data have also suggested that releasing the pressure during the rest interval between sets does not seem to acutely diminish the exercise-induced physiological responses (Freitas et al., 2019), which may therefore be performed if considerable discomfort persists. However, it may still not significantly reduce the ratings of discomfort (Freitas et al., 2020). Finally, individuals affected with cardiovascular diseases should be cautious when performing BFR resistance exercise, as previous studies have demonstrated that it may result in exaggerated cardiovascular responses, including increased blood pressure and heart rate (Pinto and Polito, 2016; Scott et al., 2018).

Therefore, considering that most individuals have currently no access to gyms or similar training facilities, or equipment needed to provide the necessary physiological stimulus to maintain skeletal muscle parameters and training status, both BFR and NLRT methods may serve as effective resistance training strategies to avoid detraining and preserve muscle mass and strength levels while the current COVID-19-related social lockdown lasts.

## Author Contributions

All authors searched the literature and wrote, revised, and approved the manuscript.

## Conflict of Interest

The authors declare that the research was conducted in the absence of any commercial or financial relationships that could be construed as a potential conflict of interest.
