# Corrigendum: Exercise: A Protective Measure or an “Open Window” for COVID-19? A Mini Review

**DOI:** 10.3389/fspor.2020.00101

**Published:** 2020-08-14

**Authors:** João B. Ferreira-Júnior, Eduardo D. S. Freitas, Suene F. N. Chaves

**Affiliations:** ^1^Federal Institute of Sudeste of Minas Gerais, Campus Rio Pomba, Rio Pomba, Brazil; ^2^Neuromuscular Laboratory, Department of Health and Exercise Science, University of Oklahoma, Norman, OK, United States; ^3^Department of Sports, Federal University of Minas Gerais, Belo Horizonte, Brazil

**Keywords:** coronavirus, immune system, endurance training, resistance training, blood flow restriction, no load resistance training

In the original article, the reference for Chen et al. (2020) was incorrectly written as Chen, P., Mao, L., Nassis, G. P., Harmer, P., Ainsworth, B. E., and Li, F. (2020). Wuhan coronavirus (2019-nCoV): the need to maintain regular physical activity while taking precautions. *J. Sport Heal. Sci*. 9, 103–104. doi: 10.1016/j.jshs.2020.02.001. It should be Chen, P., Mao, L., Nassis, G. P., Harmer, P., Ainsworth, B. E., and Li, F. (2020). Coronavirus disease (COVID-19): The need to maintain regular physical activity while taking precautions. *J. Sport Health. Sci*. 9, 103–104. doi: 10.1016/j.jshs.2020.02.001.

In the original article (Flynn et al., 1999) was not cited or it was replaced by Fahlman et al. (2000). The citation has now been inserted in the section *Can Training Status Protect Against COVID-19 Infection?*. Paragraph five should read:

The contradictions displayed across the studies discussed above may be related to differences in the immune response to training status (more trained vs. less trained individuals). Some studies have reported reductions in the lymphocyte proliferative response (Papa et al., 1989) and suppressed neutrophil function (Lewicki et al., 1988; Baj et al., 1994), whereas other studies have shown no alteration in either lymphocyte or neutrophil status after a period of exercise (Tvede et al., 1991; Flynn et al., 1999; Ferrari et al., 2013), or even increases (Brunelli et al., 2014) when comparing different training statuses.

In addition, the citation has now been inserted in the section *Can an Acute Exercise Session Increase the Risks for Covid-19 Infection?*

Paragraph two

Several studies have investigated the effects of a single exercise session on immune function by measuring acute changes in various parameters (Flynn et al., 1999; Fahlman et al., 2000; Steensberg et al., 2001; Kakanis et al., 2010). However, few studies have utilized resistance exercise protocols (Nieman et al., 1995; Flynn et al., 1999) or home-based exercises, those likely to be performed during the current pandemic which most likely consist of adapted forms of resistance exercise.

Paragraph three

Nieman et al. (1995) had participants perform multiple sets of squat exercise to failure at 65% of one-maximum repetition (1-RM) and observed conflicting results with leukocytes counts increasing immediately post-exercise and remaining elevated up to 2 h after, whereas lymphocytes number increased post-exercise but decreased below baseline levels 2 h post-exercise. Nonetheless, it is important to note that Nieman et al. (1995) utilized a sample of 10 resistance trained individuals with a mean age of ~25 years, however, COVID-19 is particularly dangerous to older individuals, which represents most of the COVID-19-related deaths reported to date (Verity et al., 2020). Therefore, it is important to consider the potential immunosuppressive effects of resistance exercise for elderly participants. In this regard, Flynn et al. (1999) investigated the acute effects of a resistance exercise session performed at 80% 1-RM on several immune function parameters in women aged 67 and 84 years and reported no suppression of immune function during the recovery period from the exercise bout. Finally, it is important to highlight that URTI incidences were not assessed in any of these studies.

Paragraph five

The different results across these studies may be due to the variability in the immune system responses to an acute exercise session. Although a number of studies have observed an immunosuppressive response after prolonged and intense exercise bouts (Steensberg et al., 2001; Kakanis et al., 2010), no changes in immune function have also been observed (Flynn et al., 1999).

In the original article, there was an error. Incorrect references were cited.

A correction has been made to the section *Can Training Status Protect Against Covid-19 Infection?, paragraph three*:

On the other hand, other studies have shown no protective effects of exercise training programs on URTI incidences (Nieman et al., 1990; Kostka and Praczko, 2007; Kostka et al., 2008; Walsh et al., 2011). To illustrate, a study with obese sedentary women who completed 15 weeks of endurance training (walking at 60% heart rate reserve), five times a week, reported no differences in the number of URTI in comparison to the untrained control subjects (Nieman et al., 1990). However, the number of days with URTI symptoms was lower in the exercise group in comparison to the control group, which was confirmed by additional studies (Kostka and Praczko, 2007; Kostka et al., 2008; Walsh et al., 2011). An additional study did not find any relationship between perceived physical fitness levels and URTI incidences (Kostka et al., 2008).

The authors apologize for these errors and state that this does not change the scientific conclusions of the article in any way. The original article has been updated.
